# Food Choices and Coronary Heart Disease: A Population Based Cohort Study of Rural Swedish Men with 12 Years of Follow-up

**DOI:** 10.3390/ijerph6102626

**Published:** 2009-10-12

**Authors:** Sara Holmberg, Anders Thelin, Eva-Lena Stiernström

**Affiliations:** 1 Research and Development Centre, Kronoberg County Council, Box 1223, SE-351 12 Växjö, Sweden; 2 Department of Public Health and Caring Sciences, Family Medicine and Clinical Epidemiology Sections, Uppsala University, Box 564, SE-751 22 Uppsala, Sweden; E-Mails: atheling@wgab.se (A.T.); evalena.stiernstrom@externa.lul.se (E-L.S.)

**Keywords:** cardiovascular disease, diet, nutrition, fruit and vegetables, dairy fat, prospective cohort study, farmers, Nordic nutritional recommendations

## Abstract

Coronary heart disease is associated with diet. Nutritional recommendations are frequently provided, but few long term studies on the effect of food choices on heart disease are available. We followed coronary heart disease morbidity and mortality in a cohort of rural men (N = 1,752) participating in a prospective observational study. Dietary choices were assessed at baseline with a 15-item food questionnaire. 138 men were hospitalized or deceased owing to coronary heart disease during the 12 year follow-up. Daily intake of fruit and vegetables was associated with a lower risk of coronary heart disease when combined with a high dairy fat consumption (odds ratio 0.39, 95% CI 0.21–0.73), but not when combined with a low dairy fat consumption (odds ratio 1.70, 95% CI 0.97–2.98). Choosing wholemeal bread or eating fish at least twice a week showed no association with the outcome.

## 1. Introduction

Coronary heart disease is linked to various lifestyle factors such as diet, physical activity, smoking and stress [1,2]. Current evidence supports an association between a limited number of dietary factors and patterns with coronary heart disease [3–5]. Consumption of fruit and vegetables seem to be heart protective [6–8] and adherence to a “Mediterranean diet” decreases the risk of coronary heart events and mortality [9,10]. However, what factors mediate the preventive effects is still a matter of debate, although the high content of fruit and vegetables and a beneficial fat profile are probable protective components [11]. The diet-heart hypothesis from the 1950s stating that saturated fats lead to heart disease via blood lipid derangement is under re-evaluation [12–16].

The Nordic nutritional recommendations are in line with international recommendations for the general public [17]. The current Swedish guidelines can be summarized in four statements: 1. Eat fruit and vegetables daily. 2. Eat bread with every meal, chose wholemeal bread. 3. Eat less saturated fat, chose liquid margarine or oils for cooking. 4. Eat fish several times a week.

We have investigated the effects of food choices on coronary heart disease over 12 years of follow-up in a cohort of Swedish farmers and non-farming rural men. The study was initiated in 1989, with the intention of studying health promoting factors related to farming. Farmers appear to have a low risk of cardiovascular disease [18–21]. Our aim was to describe food choices in this population, the degree of compliance with the Nordic nutritional recommendations and to study how reported food choices impacted on coronary heart disease.

## 2. Methods

### 2.1. Study Population

From the Swedish National Farm Register, all male farmers born between 1930 and 1949 in nine rural municipalities in Sweden were identified. The areas were chosen with consideration to known east-west and north-south cardiovascular disease gradients in the Swedish population [22] and to represent a variety of farm types and geographical variation over the country. Farmers were defined as men who owned or rented a farm and who spent at least 25 hours per week farming. The occupational activity was checked with local representatives from the Federation of Swedish Farmers. Farm labourers were not included. To each farmer a rural referent, matched by age, sex and residential area, was sampled from the National Population Register. The referents were to be occupationally active in other than farming according to the most recent census. Owing to limited non-farming populations in some of the parishes included the non-farmers were somewhat fewer than the farmers.

Altogether 1,220 farmers and 1,130 non-farmers were eligible and included in the cohort. These 2,350 men were invited to participate in an extensive health survey including questionnaires, interviews, physical examinations and laboratory test. The participation rate was 75.8 percent, with 1,782 men participating in the baseline survey in 1990–91 [23–25]. Thirty men reporting previous hospitalization for coronary heart disease at baseline were excluded, leaving 1,752 men for analyses. Seventeen men who died after 2001, but before a second survey in 2002–03 were included in total mortality rates, but no causes of death were available for these fatalities.

The study was approved by the Research Ethics Committee at the Karolinska Institute in Stockholm, Sweden and by the Regional Ethics Board, Uppsala, Sweden. All men who participated in the health survey gave their informed consent.

### 2.2. Outcomes

Number of fatalities and causes of death from 1989 through 2001 were obtained from the National Cause of Death Register. Diagnoses were according to The International Classification of Diseases (ICD), 9^th^ edition, Swedish version, 1989 through 1996 and 10^th^ edition, Swedish version, 1997 through 2001 [26,27].

Diagnoses for admissions to hospitals for the years 1990–2002 were obtained from the Hospital Patient Register, which registers all hospital admissions in Sweden with main diagnosis and up to seven additional diagnoses. We used all available diagnoses and counted the number of individuals who were given each diagnosis at least once. Cardiovascular disease was defined as ICD-9 codes 390–459 and ICD-10 codes I00–I99 and coronary heart disease was defined as ICD-9 codes 410–414 and ICD-10 codes I20–I25.

### 2.3. Food Choices

Specific food choices were assessed in a 15-item questionnaire answered at the baseline survey. One question assessed intake of fruit and berries (nearly every day, several times a week, once a week or seldom/never) and one question assessed intake of vegetables, legumes or root vegetables, except potatoes, with the same alternatives as for fruit. These items were then combined into one variable for fruit and vegetables dichotomized as daily versus less than daily intake.

Dairy fat intake was assessed by combining three questions relevant to Swedish eating habits namely usual spread on sandwiches (butter, low fat margarine or no fat), intake of whipping cream, also in sauces (daily, sometimes during the week or seldom/never) and type of milk normally consumed (non-homogenized farm milk, full fat milk with 3.0 percent fat, semi-skimmed with 1.5 percent fat or skim milk with 0.5 percent fat). Low consumption of dairy fat was defined as milk with 1.5 percent fat or less, no butter and seldom or never intake of cream. All others were denoted high consumption of dairy fat.

The most common type of bread consumed was asked about in one question with four alternatives (white bread, rye bread, whole grain rye bread and crisp/hard bread). The latter two were chosen to indicate intake of wholemeal bread. Consumption of fish was assessed using a question with four alternatives (at least once a month, at least twice a month, at least once a week and at least twice a week) and dichotomized on at least twice a week versus more seldom.

### 2.4. Baseline Characteristics

Weight and height were measured with standard procedures at the baseline survey and body mass index calculated as weight in kilograms divided by height in meters squared. Blood pressure was measured twice with mechanical blood pressure equipment (Trim line LIC^®^) after five minutes of supine rest, and the average recorded. Non-fasting blood samples were drawn, centrifuged, and refrigerated at 4 °C at the examination site and transported to the same laboratory in Uppsala for analyses within three days. The serum low-density lipoprotein was calculated using standard procedures.

Smoking habits were assessed in interview and dichotomized as current daily smoking versus no smoking. Physical workload was determined in a structured interview according to the Edholm activity scale in which the participants estimated the average number of hours working in a sitting or standing position with a moderate, heavy, or very heavy workload during an average working day [28]. Owing to large seasonal variation of workload, the farmers were asked to estimate their average workload over a longer period when needed.

### 2.5. Statistical Analyses

Power calculations were performed to ensure that the cohort would be large enough to identify significant differences in cardiovascular morbidity between subgroups over a 10-year period. The internal non-response rate was below two percent for all included variables. All participants were tracked for outcomes in the national registers. Data analyses were performed using SPSS^®^ version 14.0. A significance level of 0.05 was considered to indicate statistical significance and all test were two-tailed.

Analyses of associations between coronary heart disease and food choices were performed with logistic regression models and the results presented as odds ratios (OR) with 95 percent confidence intervals (95% CI). Models were adjusted in two steps, first with food choices adjusted for each other and second taking confounders into account. In the final model with backward elimination of variables, a p-value < 0.10 was required to stay in the model.

A significant interaction between intake of fruit and vegetables and dairy fat was revealed (Wald test, OR 3.8, p = 0.006 for interaction term). In order to illustrate the effect modification, a combined variable with four categories was constructed. [29] The category “Not fruit and vegetables daily and high intake of dairy fat” (=no exposure) was used as reference group. This allowed for effect estimates for the individual effects of both exposures of interest (daily fruit and vegetable intake and low dairy fat intake) and the joint effect. No other significant interactions between the included food choices were identified.

## 3. Results

Of the 1,752 men, 88 died during follow-up, 335 were hospitalized or died due to cardiovascular disease and 138 were hospitalized or deceased due to coronary heart disease (Table 1). All the baseline characteristics in Table 1, except physical workload were significantly associated with coronary heart disease. One third of the participants reported eating fruit and vegetables daily (Table 2). Twenty percent had low consumption of dairy fat. Seventy percent chose mostly wholemeal bread and seven percent ate fish at least twice a week. Small differences in baseline characteristics, except for serum low-density lipoprotein, were found across food choices (Table 3). Men with a low intake of dairy fat had higher body mass index, higher systolic blood pressure, lower physical workload, and were less frequently farmers and smokers.

Fruit and vegetables daily was associated with lower odds of coronary heart disease in crude analyses, and low consumption of dairy fat was associated with higher risk of coronary heart disease (Table 4). In the adjusted models, no effect of low dairy fat intake with regard to coronary heart disease was seen among men who did not eat fruit and vegetables daily (Table 5). However, men who ate fruit and vegetables daily had a lower risk of coronary heart disease if they reported a high intake of dairy fat (OR 0.39, 95% CI 0.21–0.73), but a tendency towards higher risk if combined with low dairy fat intake. Wholemeal bread or fish at least twice a week showed no association with coronary heart disease in either crude or adjusted analyses.

Farmers developed less coronary heart disease than non-farmers (crude OR 0.68, 95% CI 0.48–0.96). Since farming and physical workload were strongly correlated covariates, only one of them could be entered in the final regression model. The result was independent of whether farmer or physical workload was included, so the latter was chosen in order to better reflect the variation of physical activity among the participants.

Educational level and marital status were considered as potential socio-economic confounders and included in alternative models (data not shown). Fruit and vegetables daily and fish at least twice a week were associated with educational level and marital status. Low intake of dairy fat was associated with educational level but not with marital status. Neither educational level nor marital status was associated with coronary heart disease. The results of the final model remained virtually unchanged after these socio-economic covariates were added.

Cox proportional hazard regression with time to coronary heart disease event was performed, and revealed similar results as the logistic regression analyses (data not shown). A Kaplan-Meier analysis showed that men reporting daily intake of fruit and vegetables and a high intake of dairy fat lived on average 2 months longer than men in either one of the other three categories.

## 4. Discussion

We identified a significant interaction between intake of fruit and vegetables and dairy fat consumption. Daily fruit and vegetables consumption was associated with a lower risk of coronary heart disease only when combined with a high intake of dairy fat. Low intake of dairy fat, choosing mostly wholemeal bread or eating fish at least twice a week was not associated with a reduced risk of coronary heart disease in this cohort of rural middle-aged men. The current nutritional recommendations were hence only partly supported.

Numerous bioactive compounds present in fruit and vegetables have beneficial health effects [30] but so far no clear evidence exists for preventive health effects from extra intake of vitamins or other dietary supplements [3,31]. High intake of bioactive substances in foods in addition to a fat profile with a high and balanced intake of essential fatty acids is believed to explain the health protective effects demonstrated by the Mediterranean diet [11]. Many vitamins and other essential substances are fat-soluble, which might explain our finding of a preventive effect of daily fruit and vegetable intake only for individuals with a high dairy fat intake.

In the prospective population-based Malmö Diet and Cancer Study, total fat and saturated fat were not associated with cardiovascular events [14]. Evidence support harmful effects of *trans*-fatty acids on coronary heart disease, but there is insufficient evidence of associations between saturated fat and heart disease [3]. We lack a clear understanding of the complex effects on health of fats in relation to other components in dairy products [32,33]. A recent review and meta-analysis of 15 cohort studies of vascular disease and milk and dairy consumption found lower relative risks of stroke and/or heart disease in subjects with a high milk and dairy consumption relative to the risk in subjects with low consumption [34].

We performed the analysis also with all cardiovascular disease as outcome with the same main result. However, our focus was on coronary heart disease since the wide spectrum of cardiovascular disease includes several diagnoses not discussed in relation to nutrition.

We supplemented our analyses with stratified analyses of dairy fat. Men with the highest intake of dairy fat (butter and cream and full fat milk) had the same risk of coronary heart disease as men with a medium intake of dairy fat. Therefore the medium and high fat consumption groups were combined and denoted high consumption of dairy fat.

Many farmers and occasional rural living non-farmers in Sweden drink milk directly from the farm, i.e., non-pasteurized and non-homogenized farm milk. The effect of high intake of dairy fat found in our analyses might be related to the use of farm milk and not primarily an effect of fat composition. To deal with this we also performed analyses after exclusion of men who reported drinking primarily farm milk (518 men). The results of the final model including only men who consumed pasteurized and homogenized milk were basically unchanged. The protective effect seen for daily intake of fruit and vegetables was also evident among men drinking farm milk.

A gradual change of food choices over the follow-up period has occurred in the Swedish population [35]. If our results are valid, a substantial decline in dairy fat intake over the follow-up period could mean an underestimation of the risk of coronary heart disease. We have made preliminary analyses concerning changes of reported food choices over the 12 year follow-up period. The same food choice questionnaire was given at a second survey in 2002–03, to the same individuals as in 1990–91. Somewhat more men reported daily intake of fruit and vegetables in 2002–03 as compared with 1990–91, and fewer men reported use of butter, cream and full fat milk.

Our cohort is homogeneous regarding ethnicity and socio-economic variables and is not biased by the rural-urban health gradient [36]. The observational study design does not allow for conclusions of causality but provides a complement to experimental and theoretical studies on the effects of different diets. Individuals with heredity or other known risk factors might have modified their eating habits or be more inclined to report “healthy” food choices. We believe that part of this potential confounding is dealt with by adjusting for cardiovascular risk factors, although some residual confounding might still be present. The low risk of cardiovascular disease found among farmers could not be explain by traditional risk factors (blood pressure, overweight, blood lipids, smoking, and alcohol consumption) or by socio-economical or psychosocial characteristics in a previous eight year follow up of this cohort [37].

Another limitation is the use of a not previously validated food questionnaire. However, the items were non-complicated and the internal non-response rate was low, indicating no major difficulties for the participants when interpreting and answering the questionnaire. We could not adjust for total energy intake, a factor that has been associated with cardiovascular disease. [38] However, adjusting for physical workload partially covers this since a high workload generally is associated with a high energy intake.

Causality pathways explaining the associations between diet and coronary heart disease need further exploration. The inflammatory process of atherosclerosis raises the questions of what promotes inflammation and if and how dietary components promote or avert inflammation [39,40].

## 5. Conclusions

In conclusion, daily intake of fruit and vegetables combined with a medium-high intake of dairy fat was associated with a lower risk of coronary heart disease in this prospective population-based cohort of 1,752 rural men. Current official dietary recommendations for health promotion were hence only partly supported. Interactive effects of dietary components need more attention in diet-health research.

## Figures and Tables

**Table 1.** Baseline characteristics of study participants and cardiovascular outcomes (N = 1,752).

	**n**	**mean or percent**	minimum	maximum	**standard deviation**

***Baseline characteristics, 1990–91***					
Age, years	1,752	50.2	39	62	6.0
Body mass Index, kg/m^2^	1,752	26.4	18.3	44.4	3.2
Serum low-density lipoprotein, mmol/l	1,739	3.8	0.5	7.6	1.0
Systolic blood pressure, mm Hg	1,752	134	96	219	17
Physical workload, units	1,721	197	24	570	84
Farmer, %	994	56.7			
Daily smoker, %	409	23.3			

***Outcome, 2002/03***					
Mortality, total, %	88	5.0			
Cardiovascular disease, death or hospitalization, %	335	19.1			
Coronary heart disease, death or hospitalization, %	138	7.9			

**Table 2.** Food choices reported by study participants at baseline (N = 1,752).

	**n**	**percent**

**Fruit and vegetables**		
Less than daily	1,155	66.5
Daily	583	33.5

**Milk**		
High fat	838	48.0
Low fat	908	52.0

**Spread on sandwiches**		
Butter	845	48.6
Low fat margarine or no fat	894	51.4

**Cream**		
Daily or sometimes a week	1,015	58.8
Seldom or never	711	41.2

**Combined consumption of dairy fat**		
High fat (fat milk and/or butter and/or cream)	1,373	79.9
Low fat (low fat milk and no butter and seldom/never cream)	346	20.1

**Bread**		
White or rye bread	503	28.9
Wholemeal bread	1,239	71.1

**Fish**		
< twice a week	1,622	93.2
≥ twice a week	119	6.8

**Table 3.** Baseline characteristics according to reported food choices.

	Fruit and vegetables daily			Low dairy fat intake			Wholemeal bread			Fish ≥ twice a week		

	Yes	No		Yes	No		Yes	No		Yes	No	
			
	(n = 1,155)	(n = 583)		(n = 346)	(n = 1,373)		(n = 1,239)	(n = 503)		(n = 119)	(n = 1,622)	

	mean/%		p^1^	mean/%		p^1^	mean/%		p^1^	mean/%		p^1^

Age, years	50.1	50.2	0.687	50.7	50.0	0.057	50.6	49.2	**<0.001**	50.9	50.2	0.180
Body mass index, kg/m^2^	26.5	26.3	0.227	26.9	26.2	**<0.001**	26.4	26.3	0.620	26.2	26.4	0.563
s-Low density lipoprotein, mmol/L	3.77	3.86	0.087	3.82	3.83	0.878	3.82	3.86	0.449	3.91	3.82	0.323
Systolic blood pressure, mm Hg	132.4	134.1	0.062	136.9	132.6	**<0.001**	133.6	133.3	0.688	134.0	133.4	0.528
Physical workload, units	190.9	200.2	**0.034**	168.9	204.2	**<0.001**	200.8	188.6	**0.004**	177.7	198.4	**0.010**
Farmer, %	56.3	57.1	0.752	39.3	61.3	**<0.001**	58.1	53.7	0.090	49.6	57.4	0.096
Daily smoker, %	17.0	26.5	**<0.001**	19.1	24.7	**0.027**	21.1	28.5	**0.001**	26.9	23.0	0.336

1 p-value based on independent sample t-test for continuous variables and Chi^2^-test for categorical variables.

**Table 4.** Death or hospitalization owing to coronary heart disease during 12 years of follow-up in relation to food choices reported at baseline, crude analyses.

	number	Coronary heart disease			
		number of cases	%	Crude OR^1^	95% CI^2^

Fruit and vegetables					
Less than daily	1,155	103	8.9	1	
Daily	583	35	6.0	**0.65**	**0.44–0.97**

Milk					
High fat	838	55	6.6	1	
Low fat	908	83	9.1	1.43	**1.00–2.04**

Spread on sandwiches					

Butter	845	64	7.6	1	
Low fat margarine or no fat	894	74	8.3	1.10	0.78–1.56

Cream					

Daily or sometimes a week	1,015	65	6.4	1	
Seldom or never	711	68	9.6	**1.55**	**1.08–2.20**

Combined consumption of dairy fat					

High fat (fat milk and/or butter and/or cream)	1,373	93	6.8	1	
Low fat (low fat milk and no butter and seldom/never cream)	346	40	11.6	**1.80**	**1.22–2.66**

Bread					
White or rye bread	503	40	8.0	1	
Wholemeal bread	1,239	97	7.8	0.98	0.67–1.44

Fish					
< twice a week	1,622	128	7.9	1	
≥ twice a week	119	10	8.4	1.07	0.55–2.10

1 Odds Ratio;

2 95 percent confidence interval.

**Table 5.** Death or hospitalization owing to coronary heart disease during 12 years of follow-up according to food choices reported at baseline, adjusted analyses (N = 1,663).

	Model 1^1^		Model 2^2^	
	OR	95%CI	OR	95%CI
Not fruit and vegetables daily and				
high intake dairy fat (n = 926)	1		1	
Not fruit and vegetables daily and				
low intake dairy fat (n = 177)	1.08	0.61–1.90	0.92	0.51–1.67
Fruit and vegetables daily and				
high intake dairy fat (n = 406)	**0.36**	**0.20–0.66**	**0.39**	**0.21–0.73**
Fruit and vegetables daily and				
low intake of dairy fat (n = 154)	1.63	0.96–2.77	1.70	0.97–2.98
Bread				
White or rye (n = 491)	1		1	
Wholemeal bread (n = 1,172)	0.93	0.62–1.40	0.87	0.57–1.33
Fish				
< twice weekly (n = 1,549)	1		1	
≥ twice a week (n = 114)	1.18	0.60–2.35	1.00	0.49–2.06

1 Multiple logistic regression with food choices adjusted for each other.

2 Multiple logistic regression as for model 1 plus adjustment for age, body mass index, low density lipoprotein, systolic blood pressure, physical workload, and smoking.
